# Exploring Patient Advisors’ Perceptions of Virtual Care Across Canada: Qualitative Phenomenological Study

**DOI:** 10.2196/45215

**Published:** 2023-11-23

**Authors:** Heather Braund, Nancy Dalgarno, Sophy Chan-Nguyen, Geneviève Digby, Faizal Haji, Anne O'Riordan, Ramana Appireddy

**Affiliations:** 1 Office of Professional Development and Educational Scholarship Health Sciences Queen's University Kingston, ON Canada; 2 Department of Family Medicine Queen's University Kingston, ON Canada; 3 Division of Respirology Department of Medicine Queen's University Kingston, ON Canada; 4 Division of Neurosurgery, Department of Surgery, Faculty of Medicine, University of British Columbia Vancouver, BC Canada; 5 Division of Neurology, Department of Medicine, Queen's University Kingston, ON Canada

**Keywords:** virtual care, patient-oriented research, patient advisor, remote care, telehealth, telemedicine, phenomenology, phenomenological, perception, qualitative

## Abstract

**Background:**

While virtual care services existed prior to the emergence of COVID-19, the pandemic catalyzed a rapid transition from in-person to virtual care service delivery across the Canadian health care system. Virtual care includes synchronous or asynchronous delivery of health care services through video visits, telephone visits, or secure messaging. Patient advisors are people with patient and caregiving experiences who collaborate within the health care system to share insights and experiences in order to improve health care.

**Objective:**

This study aimed to understand patient advisors’ perceptions related to virtual care and potential impacts on health care quality.

**Methods:**

We adopted a phenomenological approach, whereby we interviewed 20 participants who were patient advisors across Canada using a semistructured interview protocol. The protocol was developed by content experts and medical education researchers. The interviews were audio-recorded, transcribed verbatim, and analyzed thematically. Data collection stopped once thematic saturation was reached. The study was conducted at Queen’s University, Kingston, Ontario. We recruited 20 participants from 5 Canadian provinces (17 female participants and 3 male participants).

**Results:**

Six themes were identified: (1) characteristics of effective health care, (2) experiences with virtual care, (3) modality preferences, (4) involvement of others, (5) risks associated with virtual care encounters, and (6) vulnerable populations. Participants reported that high-quality health care included building relationships and treating patients holistically. In general, participants described positive experiences with virtual care during the pandemic, including greater efficiency, increased accessibility, and that virtual care was less stressful and more patient centered. Participants comparing virtual care with in-person care reported that time, scheduling, and content of interactions were similar across modalities. However, participants also shared the perception that certain modalities were more appropriate for specific clinical encounters (eg, prescription renewals and follow-up appointments). Perspectives related to the involvement of family members and medical trainees were positive. Potential risks included miscommunication, privacy concerns, and inaccurate patient assessments. All participants agreed that stakeholders should be proactive in applying strategies to support vulnerable patients. Participants also recommended education for patients and providers to improve virtual care delivery.

**Conclusions:**

Participant-reported experiences of virtual care encounters were relatively positive. Future work could focus on delivering training and resources for providers and patients. While initial experiences are positive, there is a need for ongoing stakeholder engagement and evaluation to improve patient and caregiver experiences with virtual care.

## Introduction

The widespread adoption of virtual care (VC) during the pandemic is here to stay, owing to its significant benefits (improved access and efficiency, reduced wait times, convenience, better user experience, economic savings, and positive environmental impact) [1-5]. For these reasons, VC is desired by patients, health care providers, and policy makers globally [5-11]. However, there is concern about safety and privacy with VC. During VR encounters, patients consult with their health care providers using digital technologies, which may include email, SMS text messaging, or audio-videoconferencing and through personal digital devices [2].

Patient safety is an important domain of the Enterprise Risk Management framework developed by the American Society for Healthcare Risk Management and is an integral component of high-quality health care [12,13]. Patient advisors provide insight into the patient safety and technology components of this framework. Inherent need with the transition to virtual care to ensure risks related to patient privacy are managed. Health care risk management is comprised of the systems and processes used to uncover, mitigate, and prevent risks in health care institutions and is crucial to the delivery of safe and high-quality patient care [13,14]. Patient engagement is an important step toward improving health care safety and quality [15]. Patient advisors (or patient partners) are people with patient and caregiving experiences who collaborate within the health care system as individuals to share their insights and experiences for improving health care [16-18]. Given their ability to offer both the individual patient perspective (from their own experience), a broader perspective of multiple patients, and the perspective of a health care support person or advocate (a unique perspective), it is important to gather their perspectives on experience with VC [17,18] to inform future VC implementation. Patient advisors are readily accessible to researchers through institutional networks and have established motivation to collaborate in initiatives that aim to improve the health care experience for patients and caregivers.

To our knowledge, research has not explored patient advisors’ perspectives on VC, including potential risks, benefits, and recommendations in Canada. In this study, we address this gap by exploring patient advisors’ perspectives across Canada on the quality of care and patient or clinical safety with VC. Our research question is: What are patient advisors’ perceptions of virtual care and potential impacts on health care quality across Canada?

## Methods

### Methodological Orientation

The research team tried to engage the patient advisors according to the principles of the Canadian Institutes of Health Research Strategy for Patient-Oriented Research (SPOR) Patient engagement framework to the extent possible [19,20]. Patient-oriented research is about engaging patients, their caregivers, and families as partners in the research process. This engagement helps to ensure that studies focus on patient-identified priorities, which ultimately lead to better patient outcomes. This study adopted a qualitative phenomenological research design [21,22]. A phenomenological approach facilitated a greater understanding of the lived experiences of patient advisors and the VC experiences of patients [23]. More specifically, this study was guided by an interpretive (or hermeneutic) phenomenological lens [24], with a focus on understanding each individual’s meaningful experience before gaining insight into the collective whole across participants. The phenomenon of interest was patient care delivered virtually. To ensure a rigorous reporting style, the COREQ (Consolidated Criteria for Reporting Qualitative Research) checklist was used [25].

### Setting and Participants

This study describes the lived experiences of patient advisors related to VC in Canada. Participants were recruited through professional networks using snowball sampling and provided informed consent.

### Data Collection

Data were collected between August and October 2020 using virtual semistructured interviews to explore participants’ experiences with VC. A patient advisor was involved in the development of the interview guide (Multimedia Appendix 1), which was adopted in other studies exploring patient experience with virtual care [26-28]. Although participants were asked to draw on their experience as patient advisors, many also reflected on their experience as patients who had participated in VC, thus including both perspectives. The virtual interviews were conducted using Zoom (Zoom Technologies) or by telephone. The interview length ranged between 27 and 67 minutes. All interviews were audio-recorded and transcribed verbatim. Participants were given the interview guide in advance. The same researcher conducted all interviews, which helped with consistency and understanding patterns. More specifically, the researcher was very familiar with the interview guide and the data often elicited from each question. This familiarity helped the researcher to develop a rapport with each participant and add prompts as necessary to enhance the conversation. The researcher also summarized key points back to each participant throughout the interview to confirm their responses as a form of member checking.

### Data Analysis

The transcripts were uploaded into NVivo (version 12; QSR International) for open coding using a thematic approach [29]. Two researchers (HB and ND) coded a sample of the data independently to ensure intercoder agreement above 90%. For this process, they coded 1 interview together to develop a preliminary codebook and ensure shared meaning for each code. They then coded another 2 interviews independently and compared their coding line by line. They agreed on the same codes and affiliated segments of text 93% of the time. For the remaining 7%, where the researchers disagreed, they discussed the coding until they reached a consensus. This process resulted in a consensus-built codebook that was used for the remainder of the coding, which started with reading each transcript. The transcripts were coded line by line, and a code was assigned to each text segment. The code represented the smallest unit of analysis. Once all transcripts had been coded, similar codes were grouped to form subthemes. Similar subthemes were grouped to form themes. Data collection and analysis occurred concurrently, thus ensuring that data collection was ongoing until thematic saturation was reached after 15 interviews. The remaining 5 interviews were analyzed and included to ensure representation across participants. The analysis process was iterative, with ongoing discussions between the research team members. The researcher leading the analysis maintained a journal where she recorded annotations related to similarities, differences between participants, and thoughts requiring further consideration.

### Research Team and Reflexivity

The lead researcher (RA) conceptualized the study design. Author HB conducted all the interviews and analyses with intercoder reliability support from a research assistant who was not involved in the study to help address potential bias. All authors were involved in manuscript preparation and knowledge translation activities. The lead researcher RA is a male neurologist with research interests in VC. HB and ND are female researchers with extensive experience in conducting educational scholarships. Further, HB and ND have previously published studies that used a phenomenological approach. GD is a female respirologist with expertise in quality improvement. FH is a male neurosurgeon and a PhD-trained medical education scholar. AO is a patient advisor and worked as a faculty member in the School of Rehabilitation Therapy at Queen’s and as a Clinical Educator in the Office of Interprofessional Education and Practice.

None of the participants were personally known to HB. Unlike other phenomenological approaches, HB did not bracket her biases or interpretations. Instead, at the beginning of the study, she identified her assumptions related to VC and possible biases given her involvement in VC research. She regularly reflected on her interpretations and understanding of VC following each interview and throughout the research process.

### Ethical Considerations

Ethical approval was received through the Queen’s University Human Sciences research ethics board (File # 6030557).

## Results

### Overview

Our study sample included 20 patient advisors (17 female participants and 3 male participants) from 5 provinces (Ontario, Newfoundland, New Brunswick, Nova Scotia, and British Columbia). No participants approached opted out of the study. No additional demographic information was collected for confidentiality reasons. A total of 6 themes were identified: qualities and experiences of effective virtual health care, experience with VC, modality preferences, involvement of others, risks, and vulnerable populations. Recommendations are embedded across themes. The themes and subthemes are identified in Textbox 1. Participant quotations are denoted by “P” in Textbox 2. Additional quotations are provided in Multimedia Appendix 2.

**Overview of themes and affiliated subthemes.**

**Qualities of effective health care**

- High quality
- Low quality

**Experiences with virtual care**

- Benefits
- Challenges
- Facilitators

**Modality preferences**

- Similarities
- Appropriateness

**Involvement of others**

- Benefits
- Challenges
- Factors for consideration

**Risks**

- Health
- Security and privacy
- Strategies to mitigate

**Vulnerable populations**

- Challenges
- Consequences
- Meeting their needs

**Quotations organized according to theme.**

**Characteristics of effective health care**

- “I think a good physician acquaints themselves with the whole story so that they understand that they’re not just diagnosing a body...but somebody who has a contextual life....” [P8]
- “Number one, for me... confident and compassionate workforce.” [P9]
- “So I went to see another doctor who was quick with the pills but didn’t make me any better.” [P1]

**Experiences with virtual care**

- “In both cases, the contact is punctual, direct, it feels personal, and I feel that the focus on what I report or ask is better than during my office visits...” [P10]
- “[There are] definitely technology issues. [I have] no experience with computers. There’s a whole sector of the population that it’s just not going to happen...” [P9]
- “...So somebody you’ve had a relationship for a long time is fairly easy to do it on a Zoom” [P13]

**Modality preferences**

- “Our personal health information is usually recorded on the EMR [Electronic Medical Record] by the doctor. Whether it’s in-person, video or phone the doctor is going to record the information in the same way, in the same software.” [P8]
- “The quality of care is exactly the same. The questions are the same, the follow-up questions are the same, the treatment plan information and disclosure is the same, the follow-up, all of it is exactly the same...” [P13]
- “If I’m going to have a regular six-month checkup and I feel just the same as I did six months before that, wouldn’t a virtual visit make a lot more sense for both of us, for the health system, [and] for the environment?” [P1]
- “They can talk to you and if they need to see you, then a virtual visit...” [P8]
- “I do think that an in-person visit gives both parties on the team the opportunity to get a better sense of each other. I mean there is that human face-to-face interaction thing that is a very human quality. We’re all pretty good at it and it’s important in the way we establish trust in our relationships...I think that’s much more easily done in person and much more effectively done in person...” [P6]

**Involvement of others**

- “So the role families play in filling in some blanks is important, but also in hearing what is being told to the patient so that they can help to make sure that things unfold as they should...” [P2]
- “A visit I had with my mother was really distressing because she had gone downhill a bit and the facility where she is we tried to do a two-way because I wasn’t allowed in...the person who runs the facility. She didn’t even know how to make a two-way call. It was kind of a serious thing and then she just said, “Well, I think we’ll just up her antidepressant.” That was done, that’s it. I just didn’t find that to be effective for me. Because of the technology piece...” [P9]
- “I think some of the benefits would be that the students learn first-hand about the advantages and challenges of virtual care, and that’s important for their future practice, especially these days.” [P3]
- “They would have to be screened. Code of ethics, perhaps. We’re going to have to have a whole different code of ethics” [P9]
- “I actually think there should be a module every year in medical school that talks about how best to do virtual care.” [P4]

**Risks**

- “I honestly can’t think of any. Some people talk about security, ‘Is the phone being tapped?’, that kind of thing. I would say 99% of the time we don’t really care and we don’t really think about it because we’ve been using [tele]phones forever...I don’t really think that there’s many risks that are there.” [P8]
- “As opposed to on the telephone or by video conference and things could get missed. I think some diseases you wouldn’t want to take that risk; that you’d want to see the patient in person.” [P15]
- “... If I was talking about something sensitive and I needed privacy in my home and I wasn’t able to get it, I might not be completely forthcoming in the appointment.” [P13]
- “I think it’s about transparency and conversations about that technology and what you’re involved in. And really in plain language...so I think that truly informed consent to conversations virtually is really important.” [P2]

**Vulnerable populations**

- “One consequence is if it’s virtual care or nothing. If somehow they get screened out that they’re not appropriate for virtual care but they can’t receive care otherwise...” [P13]
- “...I’m thinking if there could be telehealth hubs where there are rooms with laptops, and then there’s one person staffing it to help people with the setup. The computer in those booths are already set up with all the software that’s required and a video-cam that’s required, that older populations would feel more comfortable with visiting because they know the computer is set up, that they know that there’s someone to help them with it...” [P20]

### Theme 1: Characteristics of Effective Health Care

All participants described positive and negative characteristics of health care in general that they had observed in their virtual encounters. Related to high-quality health care, many participants emphasized the importance of building relationships and considering the patient as a whole rather than only their health care issue as a means of humanizing the patient. This includes understanding the bigger picture and contextual factors. Another common characteristic identified as effective was communication, which included listening to the patient and being respectful and sensitive to their needs. Additional aspects included considering possible cultural and language barriers and being compassionate, confident, and sensitive. All participants also discussed elements of low-quality health care, which included a lack of communication, lack of scientific information, overreliance on medication, lack of alternative treatment options, and stigma associated with specific illnesses. Many of these characteristics were not unique to virtual care encounters. However, sometimes the technology was an added advantage for some characteristics associated with low-quality care such as a lack of communication.

### Theme 2: Experiences With Virtual Care

Most participants described both positive and negative experiences with VC either via telephone or video. The most articulated benefit of VC included greater efficiency in relation to time and travel. Other benefits included greater accessibility, greater convenience, and more patient centered. However, despite the benefits that participants described, they all highlighted challenges. The most reported challenge was managing technology. Additional challenges included building rapport with the provider, lack of familiarity with the necessary technology, and lack of guidelines for conducting VC. There were clear tensions articulated by the participants between the potential efficiency offered by virtual care and the need for a connection between provider and patient. As described above, many participants cited improved efficiency as part of their positive experiences with virtual care; however, they also described instances of how forming connections with providers was challenging. Recommendations included a better sharing of patient experiences related to data access. A few interviewees suggested that patients have greater access to their data from the electronic health record, which stemmed from the frustrations experienced when trying to gain access to their own health data or sharing their health information across health care providers. Some participants described the frustration associated with having to describe ailments and their history at each encounter. Access to patient electronic health data should be more accessible as a means of increasing efficiency.

All participants identified key facilitators of VC, including being comfortable with technology, sharing information, being punctual, and making appointment reminders. In addition, participants emphasized the importance of an existing relationship with the provider when transitioning to virtual care. This was believed to be important due to the added difficulty of generating a rapport and relationship with the patient virtually where many cues (eg, intonation and body language) were limited or nonexistent. Many recommendations from participants related to providing education and developing policies for guiding VC delivery. Most participants suggested offering education on VC for health care providers, patients, and the general public. More specifically, they highlighted the need for users to learn what is clinically appropriate for virtual care and how to effectively use the required technology. A number of these recommendations would likely improve efficiency, which was cited earlier as a key benefit of virtual care.

### Theme 3: Modality Preferences

All participants provided comparisons between virtual and in-person care modalities. Despite there being many similarities between modalities, participants reiterated the need for in-person care to remain an option. They felt that equivalent care is sometimes provided (eg, prescription renewal). Most participants reported that the time, schedule, and content of appointments were equivalent. Few participants reported that personal health information (eg, symptoms and medications) that is documented is also the same across modalities.

Another subtheme discussed by most participants focused on the appropriateness of each modality. They reported that a certain modality was more appropriate depending on the purpose of the clinical encounter, for example, basic encounters. In addition to ongoing checkups, VC was believed to be appropriate for follow-ups, prescription renewal, reviewing laboratory results, and some physical observations facilitated through videoconferencing. In-person care was described as necessary when a physical examination was necessary to perform procedures and establish a trusting relationship. One interviewee emphasized that they would prefer an in-person appointment for the first visit as this would help to build a relationship with the provider, which may be more difficult virtually. Virtual care was also offered as a supplement to in-person care. Multiple participants reported the need for patients to still have access to in-person care as needed. Rarely was virtual care described as the only option for care moving forward. There was a lack of consensus regarding who should decide on the modality. Some participants shared that the providers should decide on the modality of the encounter, whereas others suggested that the patients should decide as they understood their needs. Findings suggest the need for a negotiation between the provider and the patient to ensure the appropriate modality is selected. Education and training for providers and patients would also help to facilitate the process of deciding on the most appropriate modality.

### Theme 4: Involvement of Others

Participants were also asked to share their perspectives on involving other individuals, such as family members, caregivers, and medical trainees, in virtual encounters. Some participants articulated concerns about their involvement. However, they reported that they would be open to involving others if their specific concerns were addressed. This included receiving prior consent and ensuring that the trainee had an adequate skill level to be involved. Despite the reported concerns, most participants preferred family member and medical trainee involvement given the evident benefits, including increased patient support, health awareness, and increased accessibility due to their understanding and support for the care plan. Participants shared some challenges of involving others in their VC appointments, such as patients’ privacy, family dynamics, scheduling, and technology issues. Most participants suggested that including family or caregivers in the VC appointments should be optional and dependent upon prior consent from the patient. In contrast, there was agreement that medical trainees should be included in virtual encounters. The most common benefit reported was providing a learning opportunity for trainees. In addition, increased attention to patients, engaging them as new agents in VC, and setting future expectations for VC were deemed important. Some challenges were also described, such as having limited exposure to VC and privacy risks due to the sharing of personal information beyond just the patient and the provider. The need to obtain the patient’s consent was highlighted as a key prerequisite step. Other factors for consideration included the skills of the trainees, disclosing their involvement at the start of the encounter as part of the introductory phase, supervising them, and facilitating introductions between all individuals present at the encounter. To ensure that trainees have the necessary skills, interviewees reported they should participate in VC training.

### Theme 5: Risks Associated With Virtual Care Encounters

Most participants discussed virtual care in conjunction with associated risks. However, they provided suggestions to mitigate potential VC risks. Despite the variety of risks identified, they suggested that the risks were minor and could be addressed through multiple strategies. Some of the risks were related to patient health, such as miscommunication, missing subtleties when diagnosing, reliance on accurate patient descriptions, and inaccurate patient assessments. Many participants mentioned the consequences of not addressing these concerns, which could result in poorer health outcomes for vulnerable populations.

Security and privacy were frequently mentioned risks. Participants were concerned about recording the virtual encounter. All participants emphasized the need for details about how the data were documented, who had access to it, and how it would be stored securely. In addition, having a private space to facilitate a comfortable and confidential conversation was another risk. Participants suggested strategies to address these risks, including patient education, using secure software, enforcing privacy laws, and maintaining transparency. To help address privacy concerns, participants recommended that new guidelines and policies be developed outlining who is involved, how the data will be managed, and the privacy provisions. One participant suggested that patients be provided with instructional materials before the encounter to help familiarize them with the technology and proactively address technology issues. Overall, the risks were not excessive and some were not unique to virtual encounters as suggested by a few participants.

### Theme 6: Vulnerable Populations

Participants described many concerns specific to vulnerable populations and VC. They were concerned that action was required to ensure that vulnerable individuals did not “slip through the cracks.” Some of the challenges were lack of access to technology, lack of private space, and navigating system barriers (eg, siloed care and lack of care continuity). Another challenge was how many different patient groups were conceptualized as being vulnerable including but not limited to those with mental illness, cognitively impaired, homeless, those with addictions, older people, and visible minorities.

Other issues included stigma, time commitment, lack of immediate support, and difficulty with reporting symptoms. Multiple participants suggested that patients needed the option for in-person care if they were unable to access care virtually. However, VC is more accessible for individuals with certain physical impairments, which may make travel more difficult. Participants shared that this was a very daunting task that needed to be actively considered when transitioning to VC and required collaboration across invested partner groups who needed to be proactive in applying strategies to support vulnerable patients, such as increasing access to technology, providing options for care type, and telehealth hubs.

## Discussion

### Principal Findings

This study highlights patient advisors’ perceptions and experiences with VC across Canada. It presents data from patient advisors across various health care settings. Participants described characteristics they associate with high-quality VC. However, they also recognized the challenges associated with VC. Despite the challenges, such as privacy concerns, technology access, digital literacy, and the lack of in-person physical examination, participants reported positive experiences with VC. All participants recommended the availability of an in-person care option to ensure that vulnerable populations with challenges accessing VC are included. However, rather than offering in-person care, especially if the patient would like to try VC, stakeholders need to facilitate technology access. Future work in VC should explore methods for providing more equitable care for all patients.

### Previous Literature

In a recent study exploring the barriers and facilitators to VC access in a geriatric medicine clinic from Canada, the importance of integrating virtual visits into outpatient care, the need for considering inequitable access to VC, and its complexity were highlighted [30]. Similar findings were observed in another study for challenges with VC in Canada [31]. Patient representatives’ perspectives on health care during the pandemic across World Health Organization regions found that telehealth is indispensable in the future but not a solution for everything [32]. Patient representatives were identified as essential connectors and influencers during the pandemic who played an important role in VC implementation.

In September 2020, the symposium “Crossing the Virtual Chasm: Rethinking Curriculum, Competency, and Culture in the Virtual Care Era” emphasized the development of the VC education curriculum and its incorporation in graduate medical education [33]. Adopting the health care professional learning environment is important to prepare the current and future workforce for transitioning to VC [34]. Participants in this study also emphasized the need for training and education of health professionals in delivering high-quality VC.

Many challenges could be addressed with clear guidance and policies specific to VC. Herzer and Pronovost [35] identified guiding principles for VC, such as having similar safety and effectiveness to in-person care. VC should be more efficient and not contribute to care costs [35]. As described in this study, participants experienced varied levels of effectiveness with VC. Some appreciated the convenience and flexibility afforded by VC when they had a rapport established with their provider, whether it was a follow-up encounter or a simple encounter. However, other participants identified factors that facilitated an increased comfort with in-person encounters, such as the first visit, if they were dealing with sensitive things, or required a physical examination. Another key factor identified relating to efficiency was that of technological literacy for the provider and the patient. Therefore, adequate education should be provided to providers and patients to ensure their comfort with technology platforms. Additionally, VC should incorporate patient preferences and ensure equity [35]. These principles may help to integrate a patient-centered approach by considering inequities and encouraging consideration of patient preferences related to the care modality. Interviewees were positive about the use of VC and did not report that the risks outweighed the benefits. However, participants emphasized the need for policy makers and providers to be proactive in addressing the risks. They also reiterated the importance of involving patient advisors in dialogue related to risks and when identifying strategies to address the risks.

Based on the findings from our study, we propose the following recommendations to health organizations for the optimal development and integration of VC in health systems: (1) improve the digital health literacy of patients by developing tailored educational content and resources about VC; (2) improve the competency of health care providers and trainees in using digital technology and providing health care services through virtual encounters or interactions; (3) develop transparent policies around data ownership, access, and sharing of health information during VC encounters; and (4) implement shared decision-making around the use of VC to ensure trust, relationship building, better health outcomes, and mitigate risks.

### Limitations

We only interviewed patient advisors; however, some of these patient advisors were also patients and regularly reflected on their experiences with virtual care as patients explaining how that impacted their patient advisor role. Some of our previous work has explored patient perspectives on VC [26] and provider perspectives [36]. Participants were sampled across 5 provinces and are not representative of all provinces and territories. Additionally, they self-selected to participate; therefore, the sample may be skewed to those interested in VC. The diversity of the patient advisors in this study is also not clearly captured in our study. Finally, the interview protocol did not explore the participants’ type of care or purpose for VC, which may limit generalizability. Despite these limitations, this study provides valuable insight into patient advisors’ experiences and their perspectives as patients within the VC environment.

### Conclusions

This study highlights patient advisors’ perceptions of VC encounters, including characteristics of high-quality VC encounters, possible risks associated with VC, and considerations to optimize the quality of VC visits. Our findings highlight important aspects of VC encounters to maintain a patient-centered approach and propose strategies to enhance the quality of VC delivery. Future research should explore patients’ efforts to inform the implementation of VC.

## Appendix

Multimedia Appendix 1 Interview guide.

Multimedia Appendix 2 Additional quotations.

## Abbreviations

COREQ: Consolidated Criteria for Reporting Qualitative Research
SPOR: Strategy for Patient-Oriented Research
VC: virtual care
