# Supplementary material for: Exploring Patient Advisors’ Perceptions of Virtual Care Across Canada: Qualitative Phenomenological Study
Source: J Med Internet Res. 2023 Nov 23;25:e45215. doi: 10.2196/45215 (PMC10704306; doi:10.2196/45215)
Supplement: Multimedia Appendix 1 [file jmir_v25i1e45215_app1.docx]

BACKGROUND

1. To start, please tell me a little bit about your role as a patient experience advisor/patient partner.

SPECIFIC QUESTIONS: How did you come to be a patient experience advisor/partner? What aspects of your position are you most passionate about? How do you think your role improves patient outcomes and experiences with regards to health care?

1. What experience(s) have you had with virtual video conference visits, if any?
   1. ***Prompts***: In what settings? What are your general impressions?

QUALITY OF CARE

1. What do you think are some of the factors that contribute to good quality health care?
2. What are your general impressions on the quality of care provided through virtual care modalities like video conferencing?
3. In what ways do you think the quality of patient care through virtual care is different than in-person care?
   1. In what ways do you think the quality of patient care **is better than** in-person visits? Why?
      1. Can you provide an example of this?
   2. In what ways do you think the quality of patient care is **the same as** in-person visits? Why?
      1. Can you provide an example of this?
   3. In what ways do you think the quality of patient care **is worse than** in-person visits? Why?
      1. Can you provide an example of this?

BENEFITS AND RISKS

1. What would you say are important factors when conducting a successful virtual visit?
2. What do you think are potential difficulties patients may experience when conducting a virtual visit?
3. What do you think are the personal risks patients take when engaging in a virtual visit? Can you provide some examples?

FAMILY/CAREGIVER INVOLVEMENT [Not related to risk]

1. What do you think are some of the benefits of involving family members or caregivers in a patients’ virtual care experience?
2. What do you think are some of the barriers of involving family members or caregivers in a patients’ virtual care experience?
3. What factors do you think need to be considered when involving family or caregivers in virtual care?

**MEDICAL TRAINEES** [Not related to risk. Included all health professionals]

1. What do you think are some of the benefits of involving medical trainees in a patients’ virtual care experience?
2. What do you think are some of the drawbacks of involving medical trainees in a patients’ virtual care experience?
3. What factors do you think need to be considered when involving medical trainees in virtual care?

VULNERABLE POPULATIONS

1. What do you think are some of the risks of transitioning from in-person visits to virtual care?
   1. Are these risks greater for some individuals than others? If so whom, and why?
      1. ***PROMPT***: What do you think are the possible consequences for individuals that cannot use virtual care?
      2. How might these consequences impact the health and well-being of individuals who cannot use virtual care?
      3. How might some of these issues be resolved?

**PRIVACY** [Not related to risk]

1. What are some of the concerns you have had regarding privacy in virtual care?
2. What do you think might be some of the privacy provisions that should be in place to ensure patient safety in virtual care?
3. Describe any concerns regarding the privacy of patients’ personal health information with virtual care. Can you provide an example of this?

CONCLUSION

1. What are some other aspects of virtual care that might concern you as a patient experience advisor?
2. What role do you think patient experience advisors/patient partners can play in addressing the risks of virtual care to patients?
   1. *Prompts:* What initiatives do you think should be taken to address the risks of virtual care? Does this require new partnerships or resources? What are some of the challenges that Patients/patient partners might encounter when addressing the risks of virtual care?
3. How might virtual care affect your activities and advocacy moving forward?
4. These are all the questions I have for you today. Is there anything else on this topic you would like to share with me?
5. Do you have any questions for me at this time?
