# Supplementary material for: Exploring Patient Advisors’ Perceptions of Virtual Care Across Canada: Qualitative Phenomenological Study
Source: J Med Internet Res. 2023 Nov 23;25:e45215. doi: 10.2196/45215 (PMC10704306; doi:10.2196/45215)
Supplement: Multimedia Appendix 2 [file jmir_v25i1e45215_app2.docx]

| Theme | Quotations |
| --- | --- |
| 1. Qualities of effective healthcare | “Being mentally ill is especially challenging for the exceedingly small number of people who live with it every day, every hour of life. It is made momentously worse by the very limited scientific information of their root causes, the limited research and development for medical interventions, and the very strong public stigma that alters a persons ability and willingness to reveal their illness - to themselves and to loved ones.” (P10)  “The other aspect of quality of care is the relay of patient appropriate information, of passing information about prognosis or reasons behind this care plan over another, reasons about waiting or trying one drug at a time, those kinds of things. Just a level of patient education that’s appropriate.” (P13)  “Because we need to continuously build trusted relationships with our caregivers and providers.” (P8)  “There were a lot of balls that I felt like the family and caregivers were keeping in the air as backup to the health care system that didn’t seem to be talking to each other.” (P2) |
| 1. Experiences with virtual care | **“**The next appointment was in June I had with my rheumatologist, my specialist. Again, it was very positive.” (P15)  “I would say that for me personally I have a great impression of virtual care and videoconferencing for appointments.” (P20)  “There’s a whole group of people who are living on the margins too, people who have challenges with the English language, people are deaf, hard of hearing.” (P4)  “This is interesting because the first thought I had was inequity in access to technology and the internet. I think that may be the most significant aspect to study and to work toward overcoming if the health care system is moving in this direction” (P3).  “The pluses, the better than is you don’t have to sit in a waiting room for six hours. That’s really atrocious and that needs to change. I’m sure that’s across the board, number one. My husband had an issue and he really appreciated having it dealt with, not having to sit in emergency. He doesn’t have a family doctor. A lot of us here don’t have family doctors. Not having to sit in emerg for six hours.” (P9) |
| 1. Comparison with in-person care | “The quality of both still require the establishment of a workable relationship, a sense of personal connection, in my view.” (P10)  “My issues, I have a blood pressure issue so I didn’t find that particularly effective at all. And it’s connected with anxiety and care giver burnout. By phone, can you tell me your blood pressure?” (P9)  “I think virtual care would require more description and awareness of body parts to communicate the issue instead of relying on the doctor being able to observe in person, or even a person being able to point exactly” (P20) |
| 1. Involvement of others | “Well, I don’t really... you know beyond the vagaries of family dynamics which you’re going to get into whether you have virtual visits or you have in-person visits” (P6).  “I think you get more of your doctor’s attention when there are two of you there. I’ve actually had doctors admit that. In my father’s case he was hard of hearing but he was very independent and when my sisters finally started going to appointments with him the doctor sort of looked at them and said, “Oh, I didn’t know he had people.” So it gives them a bit better picture of you as a person with a family, and as a person with people who care about you. So that’s a big plus” (P1).  Maybe the code of ethics for the medical profession, if that’s part of it, needs to change ^24^cause this is a change...” (P9). |
| 1. Risks | “Because I’ve had a background in health care in planning, research, delivery I’m fairly confident about privacy in health care and how it’s protected, but I don’t think the general public shares that view” (P3)  “It’s pretty much what we have talked about. Privacy issues are the system we’re using, how safe it is. I know it can’t be fail-proof but at the same time we need to do so much so that privacy and confidentiality are protected” (P7) |
| 1. Vulnerable populations | “If we go for more virtual care then the people with no Wi-Fi are not going to get equitable care” (P1).  “I would say that probably if they have the option of in-person, then it should not impact their care” (P8).  “They will likely be very difficult to care for and very expensive to provide for, if they become a special status population delegated to the old ways of the dependent health care system” (P10).  “And more like initiative to inform patients around the risks of how information is transferred” (P20). |
